# *BcAS2* Regulates Leaf Adaxial Polarity Development in Non-Heading Chinese Cabbage by Directly Activating *BcPHB* Transcription

**DOI:** 10.3390/plants14081207

**Published:** 2025-04-14

**Authors:** Cheng Jiang, Qiang Ding, Ying He, Yiran Li, Zhanyuan Gao, Entong Li, Xilin Hou

**Affiliations:** 1 National Key Laboratory of Crop Genetics & Germplasm Innovation and Utilization, Key Laboratory of Biology and Genetic Improvement of Horticultural Crops (East China), Ministry of Agriculture and Rural Affairs of China, Engineering Research Center of Germplasm Enhancement and Utilization of Horticultural Crops, Ministry of Education of China, Nanjing Agricultural University, Nanjing 210095, China; 2021204023@stu.njau.edu.cn (C.J.); 2019204024@njau.edu.cn (Q.D.); 2020204025@stu.njau.edu.cn (Y.H.); 2019204021@stu.njau.edu.cn (Y.L.); 2021204025@stu.njau.edu.cn (Z.G.); 2021204020@stu.njau.edu.cn (E.L.); 2 Nanjing Suman Plasma Engineering Research Institute Co., Ltd., Nanjing 211162, China

**Keywords:** *BcAS2*, *BcPHB*, leaf polarity regulation, non-heading Chinese cabbage, genetic transformation

## Abstract

Leaves are the primary organs for plant photosynthesis, and their flat, symmetric morphology is crucial for plant growth and development. The LBD family transcription factor *ASYMMETRIC LEAVES 2* (*AS2*) plays a central role in the establishment of leaf polarity. In this study, we cloned the *BcAS2* gene from the non-heading Chinese cabbage cultivar “NHCC001” and successfully generated overexpression strains through genetic transformation. Phenotypic analysis revealed that overexpression of *BcAS2* led to significant upward curling of leaves in non-heading Chinese cabbage. Additionally, we found that the expression of *BcPHB*, a gene associated with leaf adaxial polarity development, was significantly up-regulated in BcAS2-overexpressing plants compared to controls. This interaction was further confirmed through yeast one-hybridization (Y1H), dual-luciferase reporter assays, and electrophoretic mobility shift assay (EMSA), all of which demonstrated that *BcAS2* directly binds to the GATA-motif site of the *BcPHB* promoter and promotes its transcription. Functional validation via overexpression and silencing of *BcPHB* confirmed its role in regulating adaxial polarity development in non-heading Chinese cabbage leaves. This study elucidates the molecular mechanism of the BcAS2-BcPHB pathway in regulating leaf polarity in non-heading Chinese cabbage, providing a theoretical foundation for morphological improvement breeding.

## 1. Introduction

Leaf morphology plays a critical role in light energy capture and conversion, particularly under low-light conditions, and is recognized as the foundation for plant growth and a direct determinant of crop yield and quality. Leaf morphogenesis is an intricately regulated process governed by the coordinated interaction of genetic networks and environmental factors [1,2]. Notably, leaf morphology critically influences plant architecture, with bilaterally symmetric leaves being essential for proper organogenesis. The establishment of leaf polarity—a central determinant of morphogenesis—serves as the foundation for crop architecture optimization via molecular breeding [3]. Therefore, elucidating the regulatory mechanisms governing leaf morphology is imperative for advancing molecular breeding strategies to improve non-heading Chinese cabbage cultivars.

Two distinct developmental phases have been characterized in leaf morphogenesis: the initial differentiation of leaf primordia from the shoot apical meristem (SAM), followed by subsequent expansion and maturation [4,5]. Within this developmental continuum, leaf polarity establishment emerges as a critical determinant of final morphology [6,7]. Polarity disruptions result in aberrant leaf morphologies, reduced lamina flatness, compromised physiological functionality, and ultimately decreased agricultural productivity [8,9,10].

In *Arabidopsis thaliana*, the *ASYMMETRIC LEAVES 2* (*AS2/AtLBD6*) transcription factor, a member of the Lateral Organ Boundaries Domain (LBD) family, functions as a master regulator of adaxial-abaxial polarity. *AS2* promotes adaxial cell fate specification to shape leaf morphology, with *as2* loss-of-function mutants displaying downward-curling leaves [11,12]. Mechanistically, AS2 coordinates a transcriptional network through repression of abaxial determinants (e.g., KANADI family genes) while activating adaxial factors, including Class III Homeodomain-Leucine Zipper (HD-ZIP III) proteins such as *PHABULOSA* (*PHB*), *PHAVOLUTA* (*PHV*), and *REVOLUTA* (*REV*) [13,14,15,16,17]. These *HD-ZIP III* genes display dynamic spatiotemporal expression patterns, initially enriched within the SAM before becoming progressively restricted to the adaxial domain of developing leaf primordia, where they establish polarity [18,19,20]. This observation suggests these genes contribute to polarity regulation through additive phenotypic effects [21,22]. Their conserved roles across species (e.g., *Arabidopsis*, rice, raize) underscore their fundamental importance in polarity regulation [23,24,25]. Notably, dominant gain-of-function alleles such as *phb-1d* and *rev-10d* induce extreme adaxialization phenotypes, producing needle-like, rod-shaped, or trumpet-like leaves with adaxialized internal tissues [26,27], further demonstrating the necessity of proper polarity for normal leaf development [28]. In addition, leaf curvature modulates photosynthetic efficiency by altering light distribution and gas exchange. For instance, upward-curling leaves in rice crops improve light capture under dense planting, whereas excessive downward curling restricts stomatal conductance. Moderate leaf rolling in corn reduces mutual leaf shading and increases photosynthesis in the population, thereby increasing tolerance to high-density planting [29,30,31]. Optimizing curvature is thus critical for yield potential.

Non-heading Chinese cabbage (*Brassica campestris* (syn. *Brassica rapa*) ssp. *Chinensis*) belongs to the Brassica genus within the Cruciferae family and is cultivated for its nutrient-rich leaves, which determine its commercial value through morphological characteristics [32]. Although previous studies have isolated *BcAS2* from the cultivar “NHCC001” and demonstrated its leaf-curling capacity when overexpressed in *Arabidopsis* [33], the molecular mechanisms governing *BcAS2* function in its native species remain poorly understood. Specifically, it remains unknown whether *BcAS2* regulates downstream polarity determinants or interacts with conserved HD-ZIP III family members such as *BcPHB* in non-heading Chinese cabbage.

This study addresses these knowledge gaps through the generation of BcAS2-overexpressing transgenic lines in non-heading Chinese cabbage, which exhibited pronounced adaxialization phenotypes with upward leaf curling. *BcPHB* was subsequently identified as a direct transcriptional target of BcAS2 using integrated molecular approaches. Functional validation via *BcPHB* overexpression and silencing confirmed its role in adaxial polarity regulation. These findings elucidate the BcAS2-BcPHB regulatory axis and provide molecular targets for optimizing leaf morphology in Brassica crops through breeding strategies.

## 2. Results

### 2.1. Identification and Expression Analysis of AS2 in Related Species

**Figure 1:**
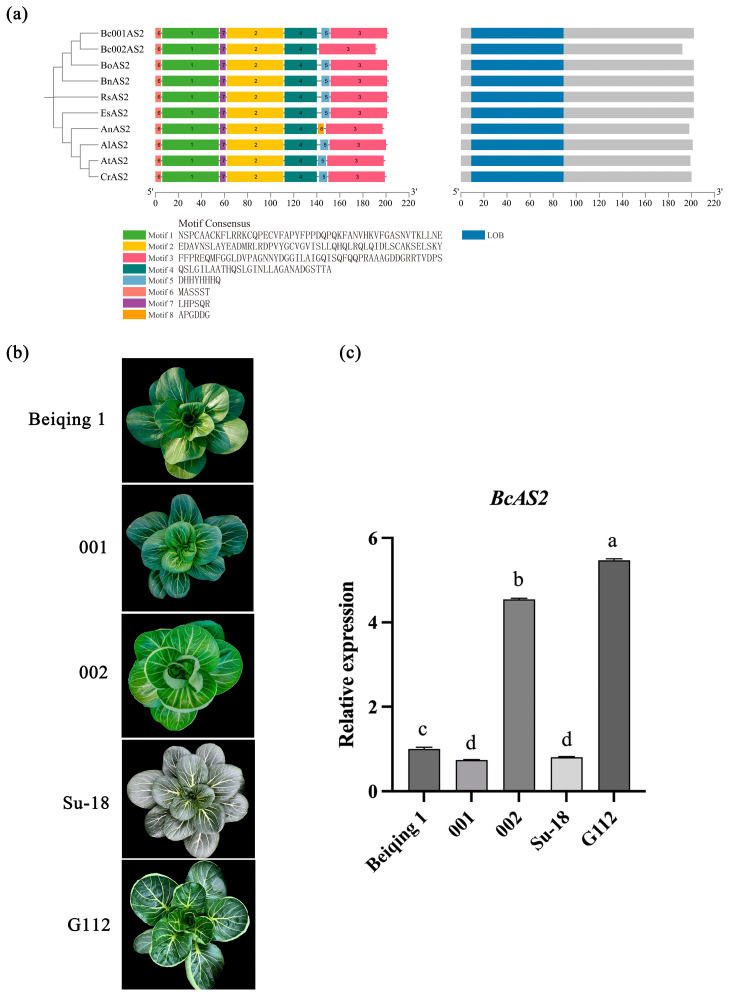
Characterization of BcAS2 protein and expression analysis in cultivars with distinct leaf polarity. (**a**) Phylogenetic analysis, multiple sequence alignment, and motif distribution of AS2 proteins across species. Sequence information is shown at the bottom. (**b**) Phenotypes of cultivars with different leaf curvature. (**c**) Relative *BcAS2* expression levels in cultivars with different leaf curvatures. Different lowercase letters denote statistically significant differences (*p* < 0.05).

Previous studies have established the essential role of the LBD family transcription factor *ASYMMETRIC LEAVES 2* (*AS2*) in regulating adaxial-abaxial polarity [11,12,34,35,36]. Sequence alignment of AS2 proteins from nine Brassicaceae species revealed that BcAS2 shares 92.3% identity with the *Brassica napus* BnAS2 (XP_013878412.1), with conserved LOB domains (Leu-35 to Gly-118) (Figure 1a). MEME analysis further identified four conserved motifs (E-value < 1 × 10^−10^) in the AS2 protein across Cruciferae species, with Motif 1 containing the characteristic LBD structural domain, defined by the sequence “CX2CX6CX3C” (Figure 1a). This suggests that AS2 is functionally relatively conserved in most cruciferae species.

To investigate the role of *BcAS2* in regulating leaf polarity in non-heading Chinese cabbage, we conducted qRT-PCR analysis in five cultivars—flat-leaved “Beiqing 1” (control), upward-curling “NHCC002” and “G112”, and downward-curling “NHCC001” and “Su-18”. The results showed significantly higher expression of *BcAS2* in the upward-curling lines and reduced expression in downward-curling lines (Figure 1b,c), suggesting that *BcAS2* promotes adaxial development.

### 2.2. Phenotypic Analysis of Leaf Polarity in BcAS2-Overexpressing Plants

**Figure 2:**
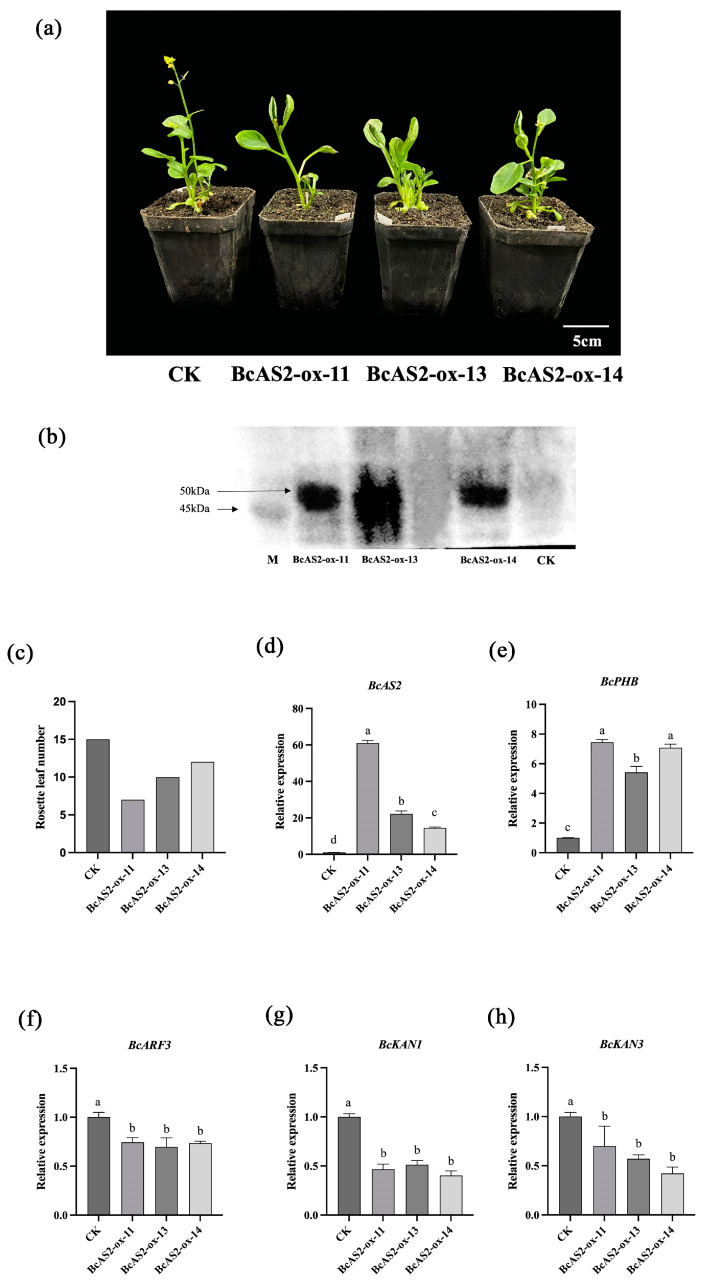
Effects of *BcAS2* overexpression on leaf polarity in non-heading Chinese cabbage. (**a**) Phenotypes of control (CK) and *BcAS2*-OE plants. (**b**) Western blot verification of transgenic lines. (**c**) Rosette leaf counts. (**d**–**h**) Expression levels of *BcAS2* and polarity-related genes. Different lowercase letters denote statistically significant differences (*p* < 0.05).

To further validate the role of *BcAS2* in the development of adaxial polarity in “NHCC” leaves, three BcAS2 overexpression lines, BcAS2-overexpressed (BcAS2-ox) (ox-11, ox-13, ox-14) were obtained by Agrobacterium-mediated genetic transformation for subsequent analyses (Figure 2a). It was further verified by qRT-PCR and Western blot experiments (Figure 2b,d). Phenotypic analysis showed that leaves of transgenic plants overexpressing *BcAS2* exhibited significant upcurling (Figure 2a), and Rosette leaf numbers decreased by about 35% (Figure 2c). In addition, we assessed the expression levels of other genes known to be involved in the regulation of leaf polarity. To elucidate the potential factors underlying the curling phenotype on the observed leaves of BcAS2 overexpressing plants, we analyzed the relative expression levels of downstream leaf polarity regulators, including *BcPHB*, *BcKAN1*, *BcKAN3*, and *BcARF3*, found in *BcAS2* overexpressing plants (Figure 2d–h). As expected, in *BcAS2* overexpressing plants, compared to control plants, qRT-PCR revealed about 5.8-fold upregulation of *BcPHB* and suppression of abaxial genes (*BcKAN1*, *BcKAN3*, *BcARF3*) in OX lines (Figure 2d–h).

### 2.3. BcAS2 Directly Activates BcPHB Transcription

**Figure 3:**
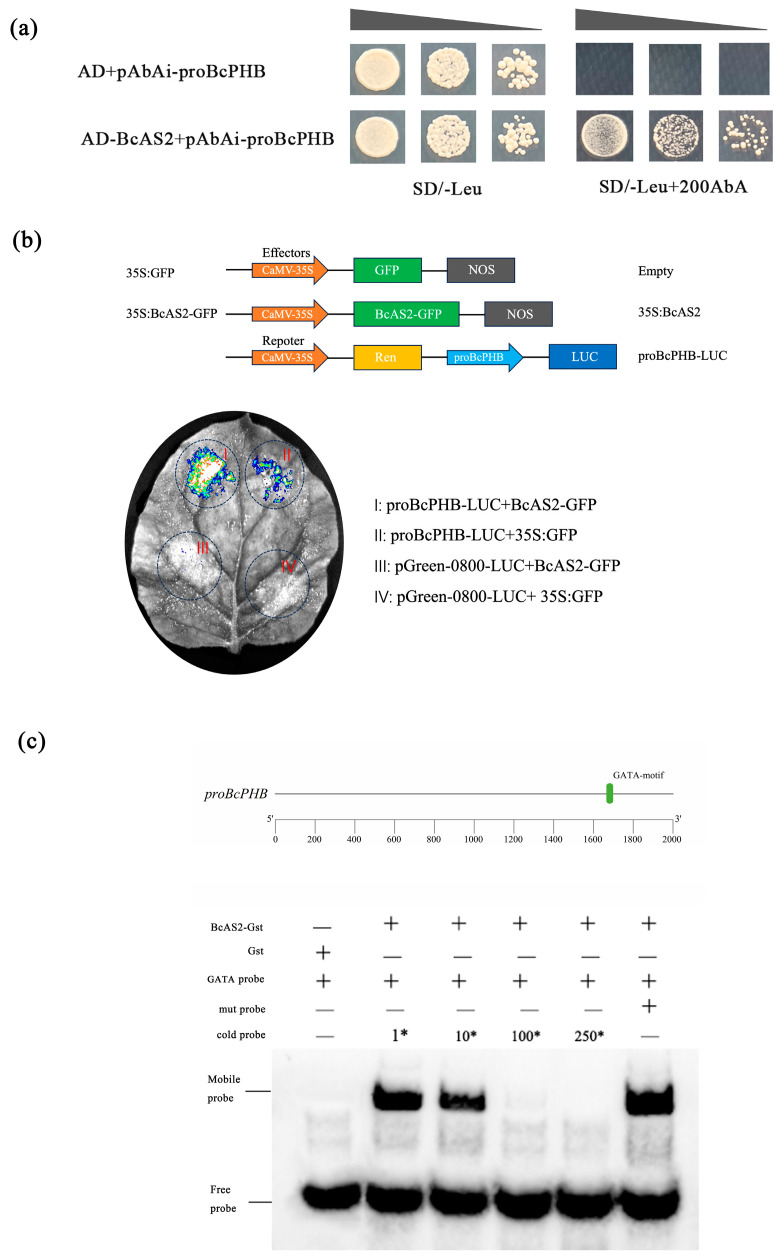
BcAS2 promotes *BcPHB* expression. (**a**) Y1H assay showing BcAS2-BcPHB promoter interaction. (**b**) Dual-luciferase assay quantifying transcriptional activation. (**c**) EMSA demonstrating BcAS2 binding to the GATA-motif. Competitor ratios (1*, 10*, 100*, 250*) are indicated.

The overexpression of BcAS2 significantly altered the expression of downstream leaf polarity regulators, which in turn affects the leaf polarity of nonbearing cabbage. Notably, BcAS2 had a significant effect on the expression level of *BcPHB*. These observations suggest a correlation between BcAS2 and *BcPHB*, which has the potential to regulate the downstream leaf polarity development pathway in Pak-choi. Yeast one-hybrid (Y1H) assays demonstrated direct BcAS2 binding to the *BcPHB* promoter (Figure 3a). The dual luciferase reporter system further confirmed that *BcAS2* could directly bind to the *BcPHB* promoter, promoting its transcription (Figure 3b). To further understand the interaction between transcription factors and promoters, the *BcPHB* promoter sequence was analyzed, and the possible binding sites for BcAS2 were predicted. Electrophoretic mobility shift assays (EMSA) confirmed sequence-specific binding to the GATA-motif (−752 to −739 bp), with binding abolished by cold competition but unaffected by mutated probes (GATA→CTCT) (Figure 3c). These findings establish BcAS2 as a direct transcriptional activator of *BcPHB*.

Based on these results, it is hypothesized that BcAS2 may regulate the development of leaf blade adaxial polarity in “NHCC” leaves through the BcAS2-BcPHB pathway. The researchers investigated the effect of *BcAS2* on *BcPHB* transcription using a dual luciferase assay. The results showed that the fluorescence intensity of *35S:BcAS2-GFP* and proB*cPHB*-luc was significantly higher than the combination of *35S:GFP* + pro*BcPHB*-luc. This confirmed enhanced transcriptional promotion of *BcPHB* by BcAS2 (Figure 3b). It was further demonstrated by EMSA experiments that BcAS2 could directly bind to the *BcPHB* promoter in vitro through the binding site GATA-motif (Figure 3c). In conclusion, BcAS2 regulates leaf polarity development by directly promoting the transcription of *BcPHB*, which promotes the development of leaf adaxial polarity.

### 2.4. Characterization of BcPHB

**Figure 4:**
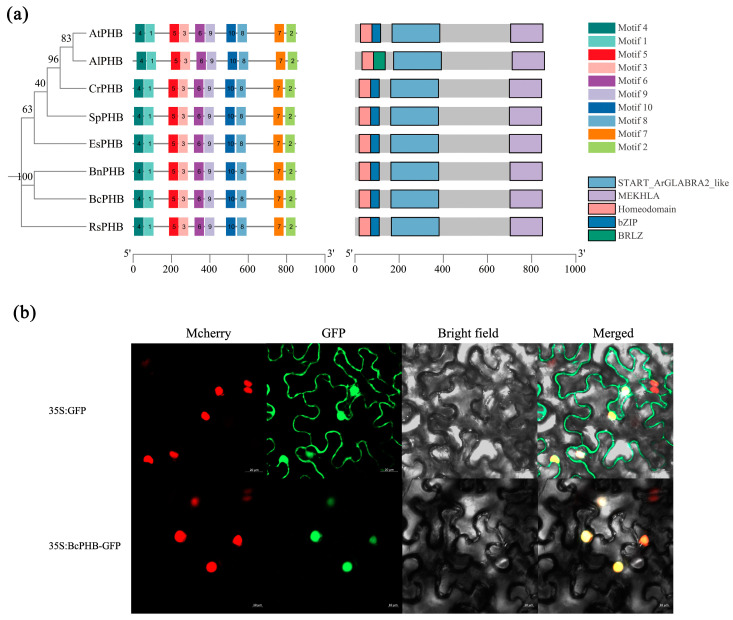
BcPHB protein features and subcellular localization. (**a**) Phylogenetic tree, sequence alignment, and motif analysis of PHB proteins. (**b**) Nuclear localization of BcPHB:GFP. Scale bar = 10 µm.

Numerous studies have demonstrated that the HD-ZIP III (Class III Homeodomain-leucine Zipper) family gene *PHB* is a key factor in the establishment of adaxial polarity in plant leaves and plays a crucial role in the regulation of leaf polarity development in a variety of crops [21]. In order to elucidate the sequence structure of BcPHB, we performed a comparative analysis of PHB amino acid sequences in a variety of horticultural and model plants. Phylogenetic analysis showed that BcPHB is closely related to BnPHB as well as to other crucifers (Figure 4a). MEME analysis showed that PHBs have the same motif composition and order in the crucifers. As shown in Figure 4a, PHB has a conserved SANT structural domain in all plants except AlPHB, suggesting that PHB is relatively functionally conserved in most plants. Analysis of the conserved structural domains showed that AtPHB (*Arabidopsis thaliana*), BnPHB (*Brassica napus*), RsPHB (*Raphanus sativus*), EsPHB (*Eutrema salsugineum*), SpPHB (*Schrenkiella parvula*), CrPHB (*Capsella rubella*), AlPHB (*Arabidopsis lyrata*), and BcPHB (*Brassica campestris* (syn. *Brassica rapa*) ssp. *Chinensis*) exhibited relatively conserved patterns, with each structural domain containing the START_ArGLABRA2_like, MEKHLA, and Homeodomain structural domains (Figure 4a). The above results indicate that PHB is functionally more conserved in Cruciferae species. The subcellular localization of BcPHB was detected by transient analysis of 35S: BcPHB-GFP, where the GFP reporter was translated and fused to the 3′ end of BcPHB cDNA. The results showed that BcPHB was localized to the nucleus (Figure 4b). In conclusion, as a member of the HD-ZIP III family of transcription factors, BcPHB is structurally and functionally similar to other PHBs in plants.

### 2.5. Expression Pattern of BcPHB

**Figure 5:**
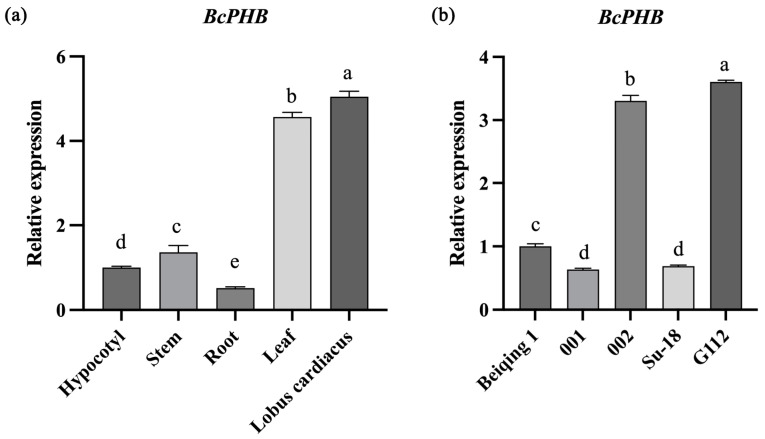
Expression Pattern of *BcPHB* (**a**) *BcPHB* expression in NHCC001 tissues. (**b**) *BcPHB* expression in five NHCC cultivars. Different lowercase letters denote statistically significant differences (*p* < 0.05).

In order to verify the expression pattern of *BcPHB* in different tissues of “NHCC”, a qPCR assay was carried out to determine its expression level using a 1-month-old sample “NHCC001”. The results showed that high *BcPHB* expression in young leaves and apical buds, with minimal expression in roots and hypocotyls (Figure 5), is consistent with its role in leaf morphogenesis [16]. In addition, *BcPHB* expression positively correlated with *BcAS2* levels across cultivars, further supporting their functional linkage (Figure 5b).

### 2.6. Phenotypic Analysis of Leaf Polarity in BcPHB-Overexpressing Plants

**Figure 6:**
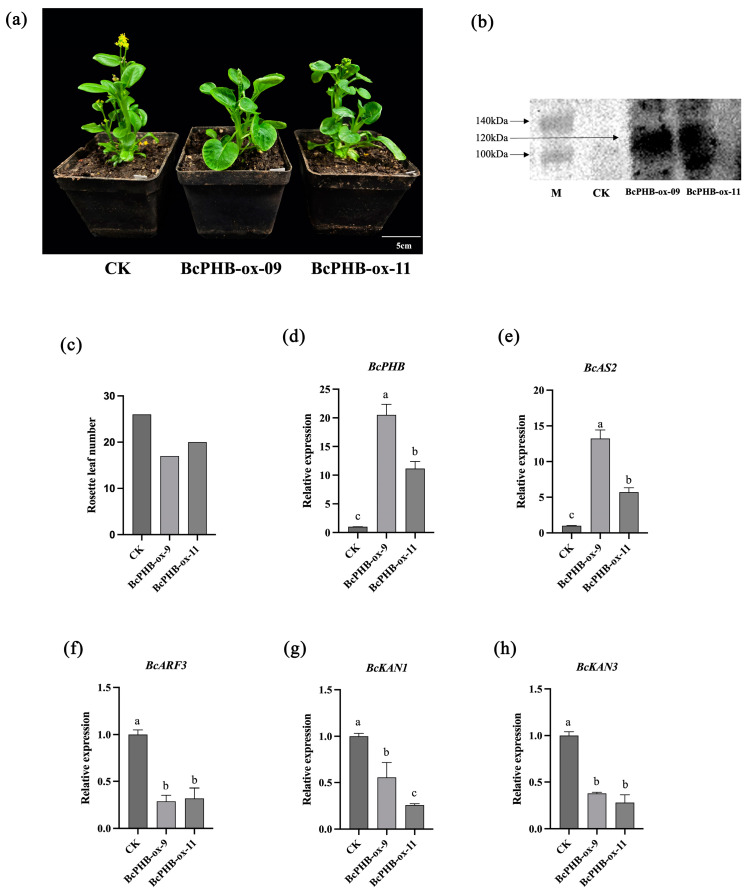
Effects of *BcPHB* overexpression on leaf polarity. (**a**) Phenotypes of CK and *BcPHB*-OE plants. (**b**) Western blot verification. (**c**) Rosette leaf counts. (**d**–**h**) Expression levels of polarity-related genes. Different lowercase letters denote statistically significant differences (*p* < 0.05).

To further validate the role of *BcPHB* in the development of adaxial polarity in “NHCC” leaves, two *BcPHB* overexpressing lines, *BcPHB*-overexpressed (*BcPHB*-ox) (ox-09, ox-11), were obtained by Agrobacterium-mediated genetic transformation for subsequent analyses (Figure 2a). It was further verified by qRT-PCR and Western blot experiments (Figure 6b,d). Phenotypic analysis showed that leaves of transgenic plants overexpressing *BcPHB* exhibited significant upcurling (Figure 6a), and the number of rosette leaves in *BcAS2*-OX plants was reduced by about 30% compared to null-loaded control plants (Figure 6c). To elucidate potential factors underlying the curled phenotype on the observed leaves of *BcPHB* overexpressing plants, we analyzed relevant leaf polarity regulators, including *BcAS2*, *BcKAN1*, *BcKAN3*, and *BcARF3*, and found that the relative expression levels of *BcPHB* in overexpressing plants (Figure 6e–h) were, as expected, significantly reduced in comparison to control plants, with the *BcPHB*-OX plants showed significantly lower mRNA levels of *BcKAN1*, *BcKAN* and *BcARF3* (Figure 6f–h). In contrast, the mRNA levels of *BcAS2* were significantly higher in *BcPHB*-OX plants than in control plants (Figure 6e).

### 2.7. Phenotypic Analysis of BcPHB-Silenced Plants

**Figure 7:**
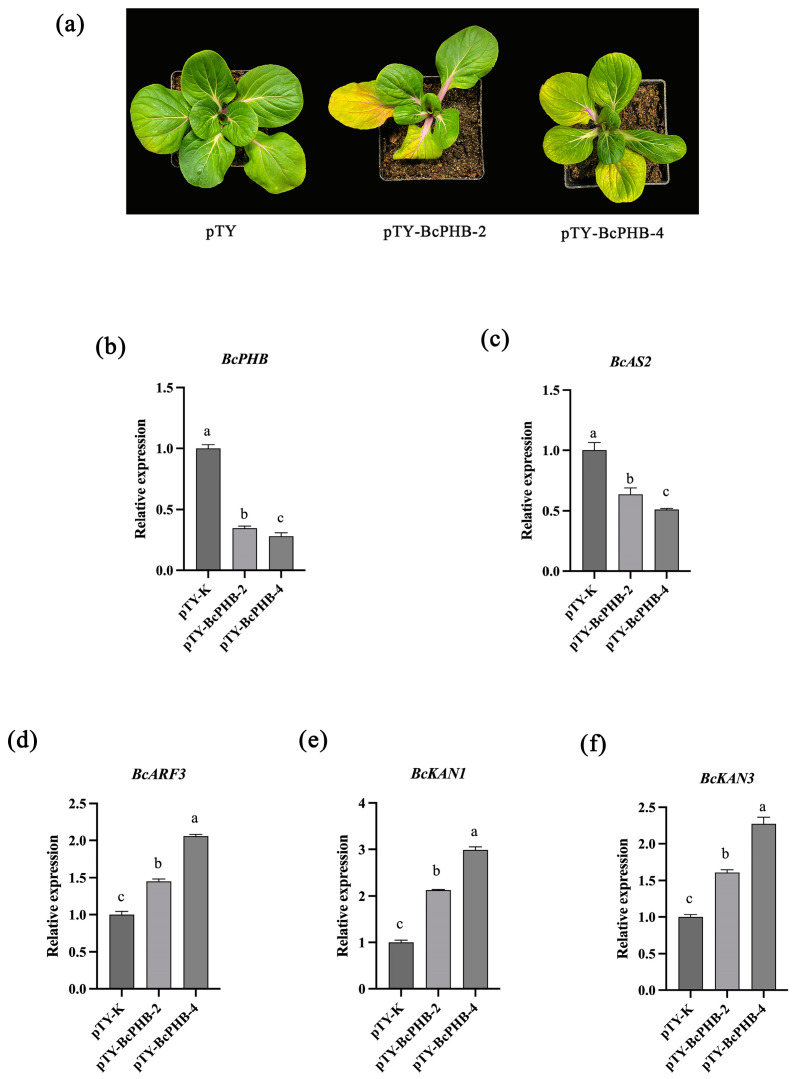
Effects of *BcPHB* silencing on leaf polarity. (**a**) Phenotypes of *BcPHB*-silenced plants. (**b**–**f**) Expression levels of *BcPHB* and polarity-related genes. Different lowercase letters denote statistically significant differences (*p* < 0.05).

To further investigate the function of *BcPHB*, we employed the virus-induced gene silencing (VIGS) technique to silence *BcPHB* in Pak-choi plants. The control plants (pTY) were injected with an empty pTY vector as a control (Figure 7a). It was observed that the leaves of *BcPHB*-silenced plants exhibited a downward curling phenotype compared to control plants (Figure 7a). *BcPHB* expression in silenced plants was reduced to about 28% of the control compared with control plants (Figure 7b). Analysis of the expression levels of *BcAS2*, *BcARF3*, *BcKAN1*, and *BcKAN3* (Figure 7c–f) revealed that *BcKAN1*, *BcKAN3*, and *BcARF3* were upregulated, while *BcAS2* was downregulated in *BcPHB*-silenced plants (Figure 7c).

## 3. Discussion

Leaf polarity establishment is a fundamental process for normal plant growth and development, governed by complex genetic regulatory networks [37]. Previous studies have found that only when the adaxial-abaxialaxis polarity is established can the leaf begin to develop normally [1,38]. Ye Lin [33] isolated the *BcAS2* gene, which has a typical LOB structural domain and is homologous to *AS2* in Arabidopsis thaliana, from the non-coccluding cabbage cultivar “NHCC001”, and initially explored its biological role in non-coccluding cabbage, but the downstream regulatory pathways have not yet been developed. The downstream regulatory pathway has not been investigated. Therefore, we further investigated the downstream leaf polarity regulators regulated by BcAS2.

Our study provides compelling evidence that *BcAS2* plays a pivotal role in regulating adaxial-abaxial polarity in non-heading Chinese cabbage leaves. Overexpression of *BcAS2* induced pronounced upward leaf curling, a phenotype reminiscent of *Arabidopsis* mutants with disrupted abaxial polarity determinants, such as *kan1 kan2* double mutants and *arf3 arf4* mutants [19,39,40]. The observed suppression of abaxial identity genes (*BcKAN1*, *BcKAN3*, *BcARF3*) and concomitant activation of the adaxial regulator *BcPHB* in *BcAS2*-overexpressing (OX) plants suggests a conserved regulatory hierarchy similar to that in *Arabidopsis*, where AS2 antagonizes abaxial-promoting factors while reinforcing adaxial fate [16,26,41,42,43].

Intriguingly, the number of rosette leaves in *BcAS2*-OX plants was reduced by about 35%, contrasting with reports in tomato (*Solanum lycopersicum*) by Xu, where overexpression of *SlAS2* increased leaf number [44]. This discrepancy may reflect species-specific subfunctionalization of AS2 orthologs. In eudicots like tomato, AS2 may primarily regulate leaf initiation through modulation of cell cycle genes such as *CYCD3* [45], whereas in *Brassica* species, *BcAS2* appears to prioritize the optimization of leaf curvature, potentially to enhance photosynthetic efficiency under specific environmental conditions. This functional divergence highlights the evolutionary plasticity of AS2-mediated regulatory networks in shaping leaf architecture across taxa. In addition, this compensatory growth pattern (i.e., reduced leaf number but increased individual leaf area in OX lines) mirrors observations in rice HD-ZIP III mutants, where prolonged SAM activity redirected resources towards post-initiation leaf expansion [46]. Such conserved trade-offs suggest a universal developmental strategy to optimize photosynthetic output under varying organ initiation rates. Further field evaluations are needed to determine if this restructuring ultimately benefits yields under agronomic conditions

Mechanistically, we demonstrated that BcAS2 directly binds to the GATA motif within the *BcPHB* promoter to activate its transcription. This interaction was rigorously validated through multiple experimental approaches: yeast one-hybrid assays confirmed physical binding, dual-luciferase reporter assays quantified transcriptional activation (3.2-fold enhancement), and EMSA resolved the specificity of BcAS2 for the GATA sequence (Figure 3). The functional significance of this regulatory axis was further corroborated by phenotypic analyses of *BcPHB*-OX and *BcPHB*-silenced plants. Overexpression of *BcPHB* recapitulated the upward-curling phenotype of *BcAS2*-OX lines, while silencing *BcPHB* via virus-induced gene silencing (VIGS) resulted in downward-curling leaves (Figure 6 and Figure 7). These reciprocal phenotypes underscore BcPHB’s central role in adaxial polarity establishment, consistent with prior studies in *Arabidopsis*, where HD-ZIP III genes like *PHB* specify adaxial identity [15,16,47,48].

Notably, we identified a feedback regulatory loop between *BcAS2* and *BcPHB*. While BcAS2 directly activates *BcPHB* transcription, *BcPHB* overexpression conversely upregulates *BcAS2* expression (Figure 6e). Conversely, *BcPHB* silencing downregulated *BcAS2* (Figure 7c). The mutual regulation of BcAS2 and BcPHB implies a stabilizing feedback loop. While yeast one-hybrid assays confirmed that BcPHB cannot directly bind to the *BcAS2* promoter (Figure S2), we propose that BcPHB indirectly stabilizes *BcAS2* expression, potentially via auxin signaling pathways [43]. However, the intermediary factors bridging this regulatory relationship remain to be elucidated.

The conservation of the AS2-PHB regulatory module across Brassicaceae is striking. Phylogenetic analyses revealed high sequence similarity between BcAS2/BnAS2 and AtAS2, with preserved LOB domains and motif architectures (Figure 1a). While motif5 is absent in NHCC-002-derived BcAS2 compared to NHCC-001, both variants preserve the intact LOB domain required for polarity regulation (Figure 1a) [12]. Complete sequence conservation within the LOB domain (motifs 1/2/7) was confirmed (Figure S1), supporting functional conservation despite motif5 divergence. Similarly, BcPHB shares conserved functional domains (SANT, MEKHLA, Homeodomain) with its *Arabidopsis* counterpart (Figure 4a), supporting functional conservation. However, subtle differences in expression patterns (e.g., tissue-specific *BcPHB* enrichment in young leaves; Figure 5) may reflect species-specific adaptations in leaf development regulation.

From an applied perspective, this study provides actionable targets for molecular breeding. The BcAS2-BcPHB pathway could be manipulated to engineer leaf curvature, a trait critical for optimizing light interception in dense planting systems. For instance, moderate overexpression of *BcAS2* or *BcPHB* might reduce mutual shading among leaves, potentially enhancing photosynthetic efficiency and yield. Conversely, targeted suppression of this pathway could benefit cultivars requiring flatter leaves for mechanical harvesting. In addition, while our data provide suggestive evidence of biomass advantages in upward-curling lines, this observation requires rigorous field validation, particularly given the documented genotype × environment interactions influencing yield-related traits mediated by leaf morphology [49].

**Figure 8:**
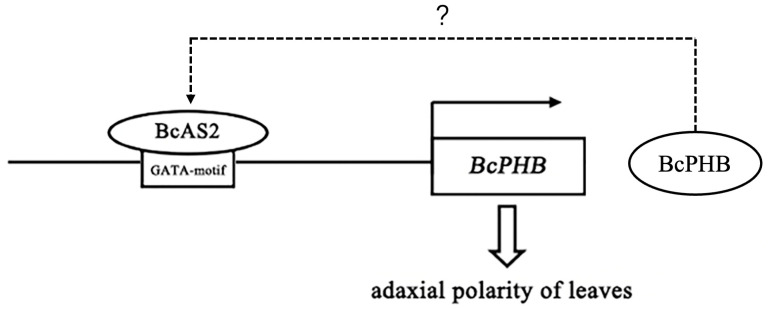
Regulatory pathway of BcAS2-BcPHB regulates adaxial polarity.

In conclusion, our work delineates a linear regulatory pathway in which BcAS2 directly activates *BcPHB* transcription to promote adaxial polarity in non-heading Chinese cabbage leaves (Figure 8). This mechanism integrates with broader polarity networks involving KANADI and ARF family genes, forming a robust system to coordinate leaf symmetry. These findings not only advance our understanding of leaf development in Brassica crops but also establish a framework for precision breeding of leaf morphology traits.

## 4. Materials and Methods

### 4.1. Plant Materials and Growth Conditions

Non-heading Chinese cabbage cultivars “NHCC001” and “49 Caixin” (*Brassica campestris* (syn. *Brassica rapa*) ssp. *Chinensis*), and tobacco (*Nicotiana benthamiana*) seedlings were cultivated in growth chambers (ZRX-380; Kesheng Experimental Instruments Co., Ltd., Ningbo, China) at 21 ± 2 °C under 16/8 h light/dark cycles (250 μmol·m^−2^·s^−1^, 40% relative humidity (RH)). Seeds of the experimental materials used in this study were were generously provided by the Laboratory of Cabbage Systems Biology at Nanjing Agricultural University, Nanjing, China. Leaf discs and plants were maintained in organic-rich substrate. Samples were flash-frozen in liquid nitrogen and stored at −80 °C.

### 4.2. Cloning and Analysis of BcPHB

*BcPHB* coding sequence (CDS) was amplified using specific primers and cloned into the pRI101-GFP vector (NdeI/KpnI sites). Homologous sequences were identified via the online BLAST tool (https://blast.ncbi.nlm.nih.gov/Blast.cgi, accessed on 15 March 2024). Multiple sequence alignment and conserved motif analysis were performed using MEGAX 7.0 and MEME website (http://meme-suite.org/tools/meme, accessed on 1 May 2024). A neighbor-joining phylogenetic tree was constructed with 1000 bootstrap replicates. Primers are listed in Table S1.

### 4.3. Subcellular Localization of BcPHB in Tobacco

*BcPHB* CDS (without stop codon) was fused to pRI101-GFP. *BcPHB:GFP* and empty *35S:GFP* vectors were transformed into *Agrobacterium tumefaciens* GV3101 (OD600 = 0.8–1.0, wavelength of 600 nm) and infiltrated into tobacco leaves. Fluorescence signals were captured after 48–60 h using a confocal microscope (Zeiss LSM 500, Oberkochen, Germany). Primers are listed in Table S1.

### 4.4. Vector Construction and Transgenic Plant Generation

Full-length *BcAS2* and *BcPHB* CDS were cloned into pRI101 to generate overexpression vectors, and pRI101 was empty as a CK control. Transgenic lines were generated via Agrobacterium-mediated transformation (GV3101 strain) of NHCC cotyledon explants [50], NHCC transgenic overexpression (OE) lines were established through tissue culture-mediated transformation, with successful transformation validated by qRT-PCR and Western blotting. Refer to (File S1) for specific methodology. Primers are listed in Table S1.

### 4.5. BcPHB Silencing via VIGS

Virus-induced gene silencing (VIGS) was performed as described in a previous study [51]. The target gene *BcPHB* 40 bp interfering fragment and its antisense sequence (ATGAACAAACTCTTGATGGAAGAGAATGATCGTCTTCAGATCTGAAGACGATCATTCTCTTCCATCAAGAGTTTGTTCAT) were inserted into the pTY vector. Gold particle-coated pTY-Empty (control) and pTY-*BcPHB* plasmids were bombarded into NHCC001 seedlings using a gene gun (1300 psi, PDS-1000/He, Bio-Rad, Hercules, CA, USA). Plants were grown under controlled conditions and sampled for qRT-PCR.

### 4.6. RNA Extraction and qRT-PCR

Total plant RNA was extracted using the RNA simple Total RNA Kit (No. DP432; Tengen Biotechnology Co., Ltd., Beijing, China), and reverse transcription of the RNA was performed using the Hifair^®^ V one-step RT-gDNA digestion Super Mix for qPCR (No. 11142ES10; Yesheng Co., Ltd., Shanghai, China) was used for reverse transcription of RNA. Samples were analyzed by quantitative polymerase chain reaction (qPCR) using Hieff^®^ qPCR SYBR Green Master Mix (No. 11201ES08; YEASEN, Shanghai, China) and CFX Connect Real-Time PCR Detection System (Bio-Rad, Hercules, CA, USA) according to the manufacturer’s instructions. Expression data were calculated using the 2^−ΔΔCt^ method with three independent replicates [52], the relative expression levels of the target genes were calculated, and the results of NHCC were normalized using *BcGAPDH* (BraC08g031360.1) as an internal reference gene [53]. Primers are listed in Table S1.

### 4.7. Yeast One-Hybrid (Y1H) Assay

The 2000 bp sequence upstream of the *BcPHB* transcriptional start site was used as the promoter region, and the amplified DNA target fragment was inserted into the pAbAi vector to obtain the bait vector pAbAi-BcPHB. The CDS, the terminatorless codon of *BcAS2*, was fused to the pGADT7(AD) vector. Yeast one-hybrid (Y1H) assays were performed following the Yeast Protocols Handbook (Clontech, San Jose, CA, USA). Bait (pAbAi-BcPHB) and prey (pGADT7-BcAS2) vectors were co-transformed into Y1H Gold yeast strains. Protein-DNA interactions were assessed on SD/-Ura/-Leu plates containing aureobasidin A (AbA) at optimized concentrations. Refer to (File S2) for specific methodology. Primers are listed in Table S1.

### 4.8. Dual-Luciferase Assay

The dual luciferase assay was performed according to the method of Wang et al. [54]. The *BcPHB* promoter sequence was inserted into the pGreen0800-LUC vector to obtain a reporter vector. The *BcAS2* stop codon-free CDS was cloned into the pRI101 vector to generate an effector vector. The vector product was then transformed into A. tumefaciens GV3101 (psoup) (Tolo Biotech, Shanghai, China). Agrobacterium cells containing the reporter gene and effector constructs were injected into tobacco leaves at a ratio of 1:9. After 48–96 h, fluorescence at the injection site was detected using a Tanon 4600 (Shanghai, China) in vivo imager. Activity levels of firefly luciferase (LUC) and Ray nira luciferase (REN) were determined using the Dual Luciferase Reporter Kit (No. 11402ES60; YEASEN) according to the manufacturer’s instructions. Primers are listed in Table S1.

### 4.9. Electrophoretic Mobility Shift Assay (EMSA)

BcAS2 was cloned into the pGEX-4T-1 (GST) vector, and the resulting recombinant plasmid was then transformed into *E. coli* (DE3) fertile cells. Oligonucleotide probes were synthesized using online resources (http://www.tsingke.net/, accessed on 23 May 2024) and labeled with biotin at the 5′ and 3′ ends. The biotin-labeled probes and mutation probes were synthesized by Shanghai Sangong Biotechnology Co. (Shanghai, China). Double-stranded DNA probes were prepared by annealing complementary oligonucleotides. The annealing process consisted of heating the samples to 95 °C for 3 min, then gradually cooling the samples from 1 °C to 25 °C at 90 s intervals and separating the bound probes from the probes on a nondenaturing acrylamide gel. The electrophoretic mobility shift assay (EMSA) was performed using a chemiluminescent EMSA kit (Shanghai Beo Tianmei Biotechnology Co., Ltd., Shanghai, China) and imaged with a protein imaging system (Tanon 4600; Tanon Science & Technology Co., Ltd., Shanghai, China). Details of the DNA probes used are shown in Table S1.

### 4.10. Statistical Analysis

Experiments included three biological and technical replicates. Statistical analyses used ANOVA with Tukey’s test (*p* < 0.05).

## 5. Conclusions

This study we findings establish the BcAS2-BcPHB pathway as a key regulator of leaf polarity in non-heading Chinese cabbage. Overexpression of *BcAS2* induces pronounced upward leaf curling by directly activating *BcPHB* transcription, which suppresses abaxial identity genes (e.g., *BcKAN1*, *BcKAN3*, *BcARF3*) and reinforces adaxial cell fate. Molecular validation through Y1H, dual-luciferase, and EMSA assays confirmed that BcAS2 binds the GATA-motif in the *BcPHB* promoter to enhance its expression. Reciprocal experiments—*BcPHB* overexpression phenocopying *BcAS2*-OX lines and *BcPHB* silencing causing downward curling—definitively link *BcPHB* to adaxial polarity regulation. Furthermore, the mutual regulatory loop between *BcAS2* and *BcPHB* highlights a stabilizing mechanism for adaxial identity. This study provides both mechanistic insights into leaf polarity establishment in Brassica crops and actionable targets for molecular breeding of leaf morphology traits.

## Data Availability

Data are contained within the article and Supplementary Materials.

## Supplementary Materials

The following supporting information can be downloaded at: https://www.mdpi.com/article/10.3390/plants14081207/s1.
