# Supplementary material for: BcAS2 Regulates Leaf Adaxial Polarity Development in Non-Heading Chinese Cabbage by Directly Activating BcPHB Transcription
Source: Plants (Basel). 2025 Apr 14;14(8):1207. doi: 10.3390/plants14081207 (PMC12030544; doi:10.3390/plants14081207)
Supplement: Supplementary file 1 [file plants-14-01207-s001.zip › File S1.pdf]

File S1: Generation of Transgenic NHCC Plants

Vector Construction and Agrobacterium Transformation

The overexpression vector pRI101-GFP was utilized for recombinant plasmid construction following the methodology described in Section 4.3. Sequence-verified recombinant plasmids were introduced into Agrobacterium tumefaciens strain GV3101 competent cells.

Plant Infection Protocol

1. Sterile Seedling Preparation

Seeds of the "49 Caixin" cultivar (Brassica campestris) were surface-sterilized in 75% ethanol under laminar flow, followed by immersion in 30% sodium hypochlorite for 10 min. After five rinses with sterile water, seeds were air-dried on autoclaved filter paper and germinated on 1/2 MS solid medium (Table S2-1) under controlled conditions: 24°C/18°C (day/night), 16-h photoperiod (4,000 lux) for 4–5 days.

2. Pre-culture

Cotyledons with 0.5-cm petioles were excised from apical meristems using sterile blades. Explants were inserted vertically into pre-culture medium (Table S2-1) for 2–3 days.

3. Co-culture

Agrobacterium suspensions were prepared as per Section 1.2.3. Pelleted cells were resuspended in 10× diluted 1/2 MS liquid medium to OD_{600} = 0.2, supplemented with 100 μM acetosyringone (AS), and incubated at 28°C (220 rpm, dark) for 2–4 h. Pre-cultured explants were immersed in the bacterial suspension for 12 min, blotted dry, and transferred to co-culture medium (Table S2-1) under darkness for 2–3 days.

4. Differentiation Culture

Explants were washed 3–5 times with sterile water containing 250 mg/L timentin (TMT) and 250 mg/L carbenicillin (Carb), air-dried, and cultured on differentiation medium (Table S2-1) for 20–30 days under standard growth conditions.

5. Subculture

Vigorous adventitious shoots were transferred to subculture medium (Table S2-1) for 3–5 weeks.

6. Transgenic Screening

Shoots were subjected to antibiotic selection on subculture medium containing vector-specific resistance markers. Non-transgenic shoots exhibited chlorosis or necrosis.

7. Rooting and Acclimatization

Transgenic plantlets were rooted on specialized medium (Table S2-1). Robust root systems were hardened by 3-day gradual lid opening, followed by transplantation to soil substrate under controlled-environment growth.

Table S2-1 Medium formulations

Medium Name	Basal Medium[*1]	Supplements[*2]
Germination	1/2 MS+30 g/l sucrose+7.5 g/l agar	-
Pre/Co-culture	MS+30 g/l sucrose+7.5 g/l agar	1.5 mg/l TDZ+0.25mg/l NAA
Differentiation	MS+30 g/l sucrose+7.5 g/l agar	2 mg/l TDZ+0.25 mg/l NAA+4.5 mg/l $AgNO_3$+250 mg/l TMT+250 mg/l Carb

Subculture	MS+30 g/l sucrose+7.5 g/l agar	3 mg/l 6-BA+0.1 mg/l NAA+5 mg/l $AgNO_3$+250 mg/l TMT+250 mg/l Carb （+10mg/l Hrg*3）
Rooting	1/2 MS+30 g/l sucrose+7.5 g/l agar	250 mg/l TMT+250 mg/l Carb+10mg/l Hrg*3

*1 pH 5.8, autoclave-sterilized;

*2 Filter-sterilized hormones/antibiotics added post-autoclaving;

*3 Hygromycin selection marker varied by vector.

Transgenic Plant Validation

2. RNA-Level Analysis

Gene expression was quantified via qRT-PCR as per Section 4.6 (RNA extraction, reverse transcription).

3. Protein-Level Verification

Protein Extraction: 0.1 g leaf tissue was ground in liquid nitrogen, mixed with 200 μL 2×SDS buffer (10% β-mercaptoethanol), heated (95°C, 5 min), and centrifuged (12,000 rpm, 5 min).

SDS-PAGE:

Separating Gel (Table S2-2):

Table S2-**2.** Separating adhesive system

**Component**	**Volume**
Separating Buffer with 0.4% SDS	1.375 mL
40% Acry/bis	1.375 mL
10% APS	50 μL
TEMED	5 μL
ddH_2O	2.68 mL

Polymerized under 1 mL isopropanol for 30 min.

Table S2-3 Concentrated rubber system

**Component**	**Volume**
Stacking Buffer with 0.4% SDS	0.75
40% Acry/bis	165 μL
10% APS	14 μL
TEMED	1.4 μL

Electrophoresis: 80 V until samples entered separating gel, then 120 V until bromophenol blue reached gel bottom.

Western Blotting:

Table S2-4 10*SDS-PAGE buffer liquid system

**Component**	**Volume（g）**
Tris	30.3
glycine	144.13
ddH20	up to 1000 ml
Total	1000 ml

Membrane transfer: The PVDF membrane was infiltrated into methanol solution for 1 min for activation, and the membrane transfer filter paper, PVDF membrane, fiber mat and gel with gel concentrate removed were put into the membrane transfer buffer to equilibrate for about 3 min. The membrane transfer solution was prepared as follows:

Table S2-5　Rotating film buffer liquid system

**Component**	**Volume（ml）**
methyl alcohol	100
10% SDS	10
ddH20	890
Total	1000

PVDF membranes were activated in methanol, equilibrated in transfer buffer, and subjected to semi-dry transfer (100 V, 60 min, 4°C).

Antibody Incubation:

Blocked with 5% skim milk (30 min), probed with primary antibody (1:1000 dilution in blocking buffer, 3 h), washed with TBST, incubated with HRP-conjugated secondary antibody (1:1000, 1–2 h), and visualized using Yisheng Biotech ECL kit (Bio-Rad ChemiDoc).
