# Supplementary material for: BcAS2 Regulates Leaf Adaxial Polarity Development in Non-Heading Chinese Cabbage by Directly Activating BcPHB Transcription
Source: Plants (Basel). 2025 Apr 14;14(8):1207. doi: 10.3390/plants14081207 (PMC12030544; doi:10.3390/plants14081207)
Supplement: Supplementary file 1 [file plants-14-01207-s001.zip › File S2.pdf]

File S2: Yeast One-Hybrid Assay

Recombinant Plasmid Linearization and Genomic Integration

The recombinant plasmid was linearized using *BstBI* or *BbsI* restriction enzymes (New England Biolabs, NEB). Digested products were electrophoresed on 1% agarose gel, and target bands were purified using a gel extraction kit.

Single Enzyme Digestion Reaction System (Table S3-1):

Table S3-1 Single enzyme reaction system

Component	Volume（μl）
BstbI/BbsI	1
Recombinant plasmid	1μg
10*buffer	5
ddH2O	Up to 50
Total	50

Linearized plasmids were transformed into Y1HGold competent cells using the following protocol:

1. **Transformation**:
   - 100 μL thawed Y1HGold competent cells were mixed with 1–5 μg linearized plasmid (≤15 μL), 10 μL heat-denatured Carrier DNA (95–100°C for 5 min, flash-cooled on ice, repeated once), and 500 μL PEG/LiAc solution.
   - Incubated at 30°C for 30 min (vortexed 6–8 times at 15 min).
   - Heat-shocked at 42°C for 15 min (vortexed 6–8 times at 7.5 min).
   - Centrifuged at 10,000 rpm for 30 s; supernatant discarded.
   - Pellet resuspended in 400 μL ddH_2O, centrifuged again, and finally resuspended in 50 μL ddH_2O.
   - Suspension was plated on SD/-Ura agar and incubated at 29°C for 72 h.
2. **Integration Verification**:

Single colonies were subjected to PCR using Matchmaker™ Insert Check PCR Mix 1 (Takara Bio) to confirm genomic integration.

PCR Reaction System (Table S3-2):

Table S3-2 PCR reaction system

Component	Volume（μl）
Matchmaker Insert Check PCR Mix 1	5
Yeast culture	0.5
ddH2O	5
Total	10

Positive clones exhibited bands corresponding to 1.4 kb + insert size.

3. **Determination of AbA Autoactivation Threshold**

Positive yeast strains were diluted to OD_{600} = 0.2 and further serially diluted (1/10, 1/100). Aliquots (10 μL) of each dilution were spotted onto SD/-Ura plates containing gradient concentrations of aureobasidin A (AbA). Plates were incubated at 28°C for 2–3 days. The minimal inhibitory AbA concentration was defined as the lowest dose preventing yeast growth.

4. **Yeast One-Hybrid Interaction Assay**

Competent cells from positive clones (Section 1) were prepared and transformed with either AD-BcARF3 or empty AD vectors using the protocol in Section 1. Transformants were plated on SD/-Leu medium and incubated at 28°C for 2–3 days.

Interaction Validation:

- Colonies were resuspended in ddH_2O to OD_{600} = 0.2, then diluted (1/10, 1/100).
- 5 μL of each dilution was spotted onto SD/-Leu plates supplemented with 150 ng/mL AbA.
- Plates were incubated at 28°C for 2–3 days. Protein-DNA interactions were confirmed by colony growth under selective conditions.
