# Supplementary figures and images for: BcAS2 Regulates Leaf Adaxial Polarity Development in Non-Heading Chinese Cabbage by Directly Activating BcPHB Transcription
Source: Plants (Basel). 2025 Apr 14;14(8):1207. doi: 10.3390/plants14081207 (PMC12030544; doi:10.3390/plants14081207)

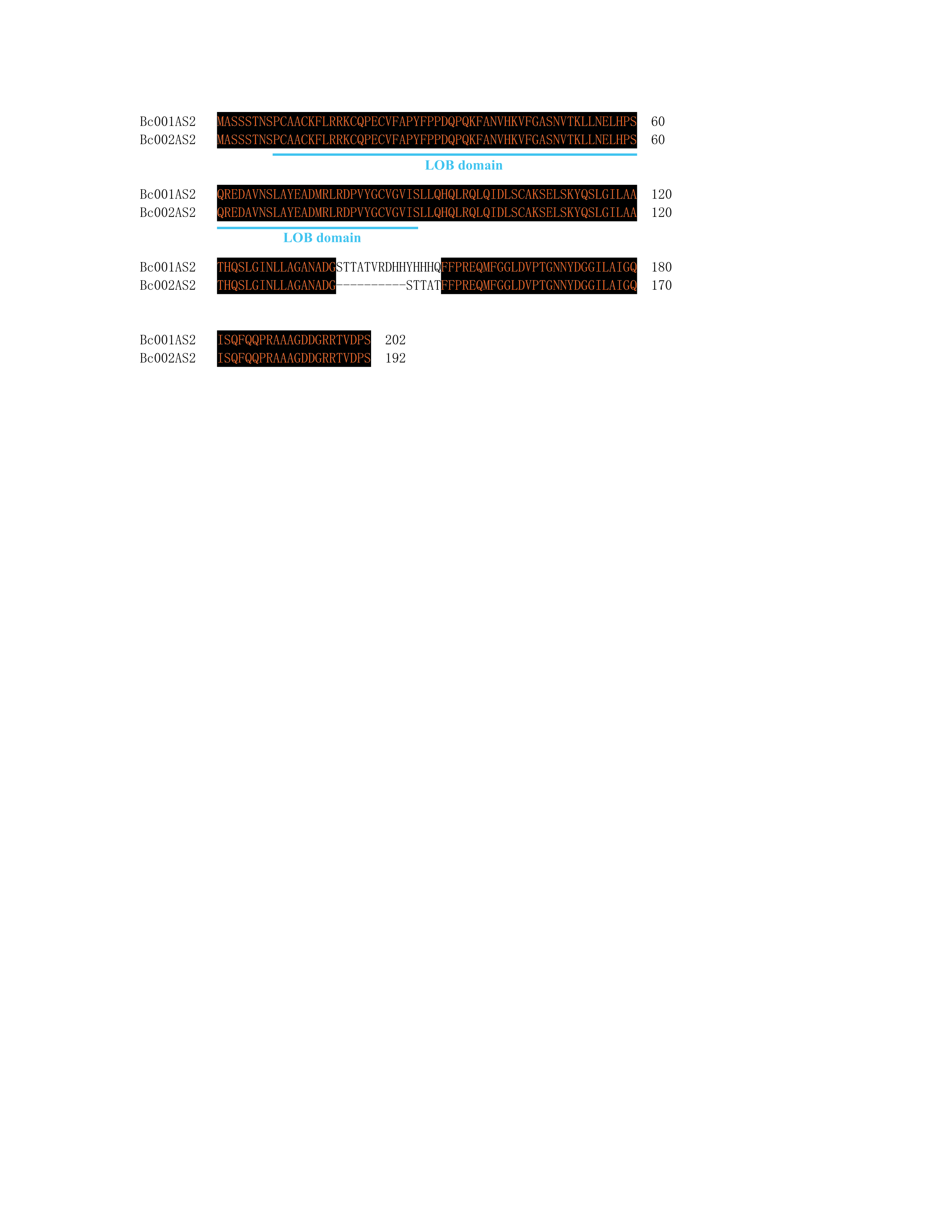

Supplement: Supplementary file 1 [file plants-14-01207-s001.zip › Figure S1.Amino acid sequence comparison of the LOB structural domains in BcAS2(001) and BcAS2(002). tif.tif]

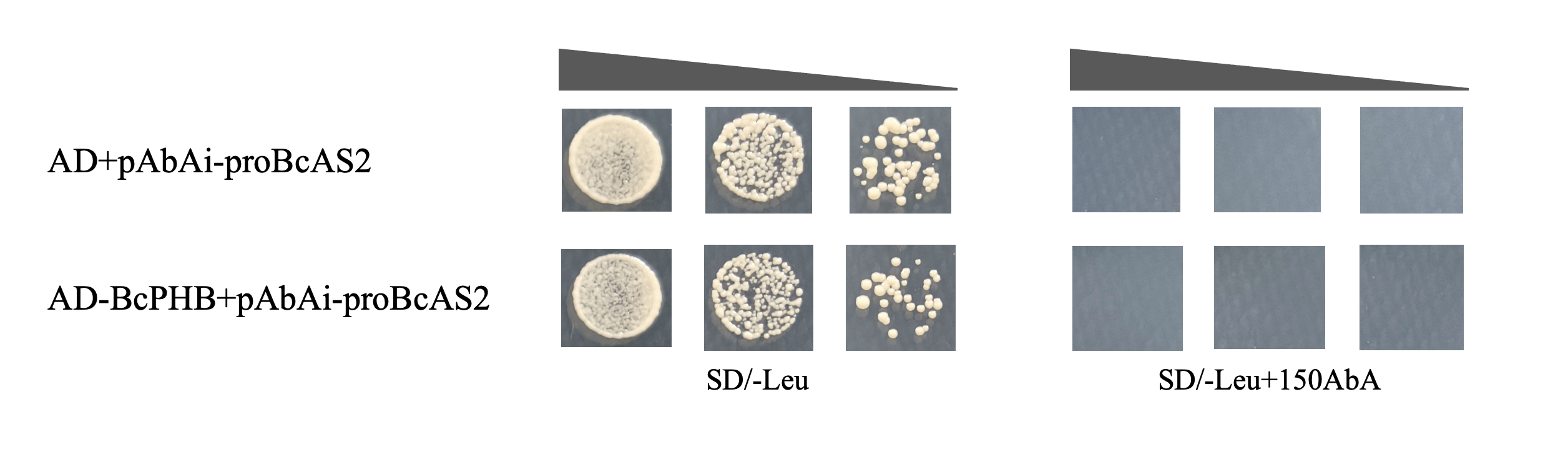

Supplement: Supplementary file 1 [file plants-14-01207-s001.zip › Figure S2.Y1H verified that BcPHB cannot bind the BcAS2 promoter.tif]
